# Supplementary material for: The Relevance of Optical Coherence Tomography Angiography in Screening and Monitoring Hypertensive Patients with Carotid Artery Stenosis
Source: Diagnostics (Basel). 2025 May 30;15(11):1393. doi: 10.3390/diagnostics15111393 (PMC12153997; doi:10.3390/diagnostics15111393)
Supplement: Supplementary file 1 [file diagnostics-15-01393-s001.zip › diagnostics-3560157-supplementary.pdf]

QQ Plot displays distribution of data, regression line, correlation type and confidence interval.

QQ Plots analyzing OCTA parameters in right eye group and right Carotid Doppler US velocities are displayed in Figures S1-8.

QQ Plots analyzing OCTA parameters in the right eye group and right Carotid Doppler US velocities.

Figure S1. Right eye QQ Plot for right ICA PSV and OCTA parameters (NFA Area, VFA Flow Area).

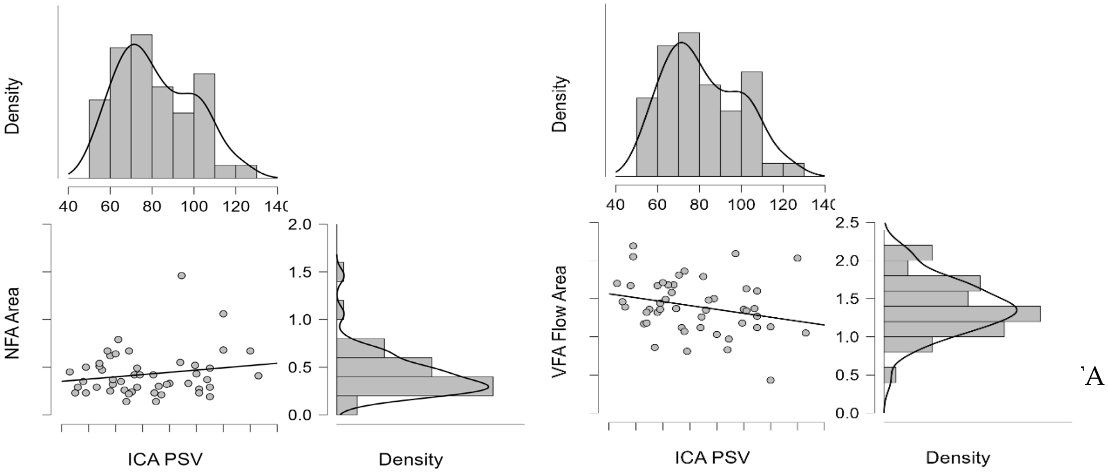

NFA: non-flow area; VFA: vascular flow area; PSV: peak-systolic velocity; ICA: internal carotid artery.

Figure S2. Right eye QQ Plot for right ICA EDV and OCTA parameters (NFA Area, VFA Flow Area).

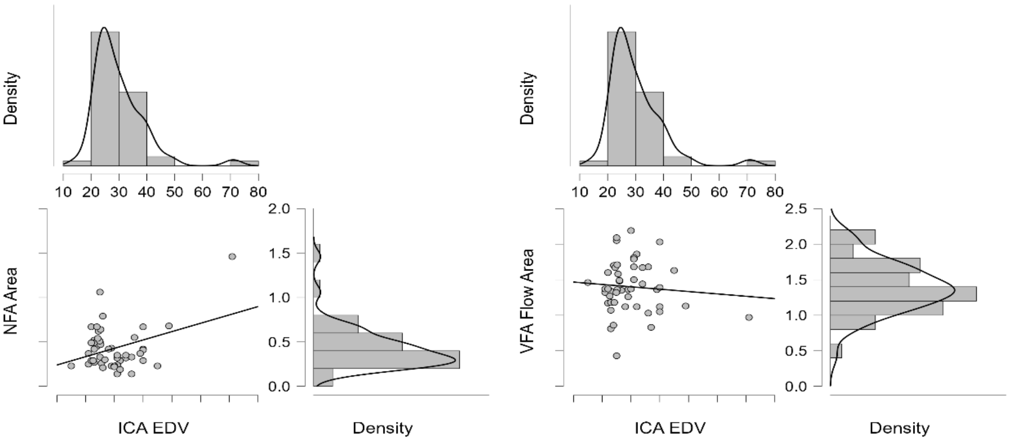

NFA: non-flow area; VFA: vascular flow area; EDV: end-diastolic velocity; ICA: internal carotid artery.

Figure S3. Right eye QQ Plot for right ECA PSV and OCTA parameters (NFA Area, VFA Flow Area).

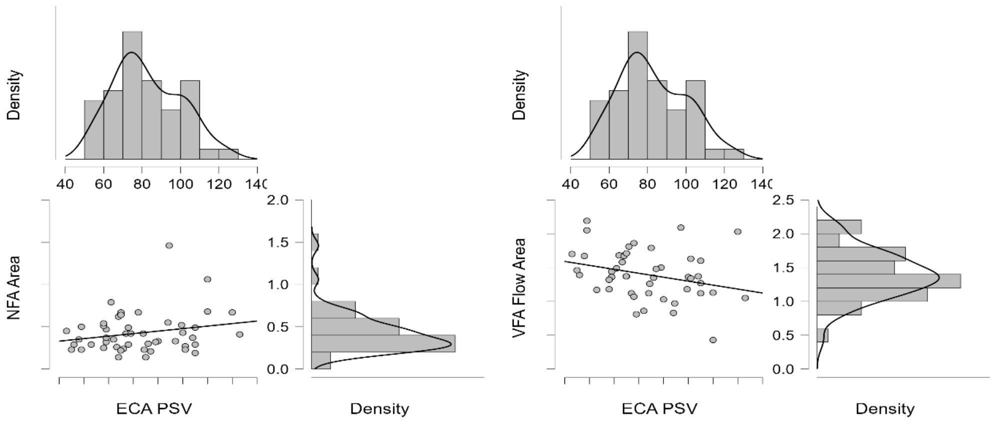

NFA: non-flow area; VFA: vascular flow area; PSV: peak-systolic velocity; ECA: external carotid artery.

Figure S4. Right eye QQ Plot for right ECA EDV and OCTA parameters (NFA Area, VFA Flow Area).

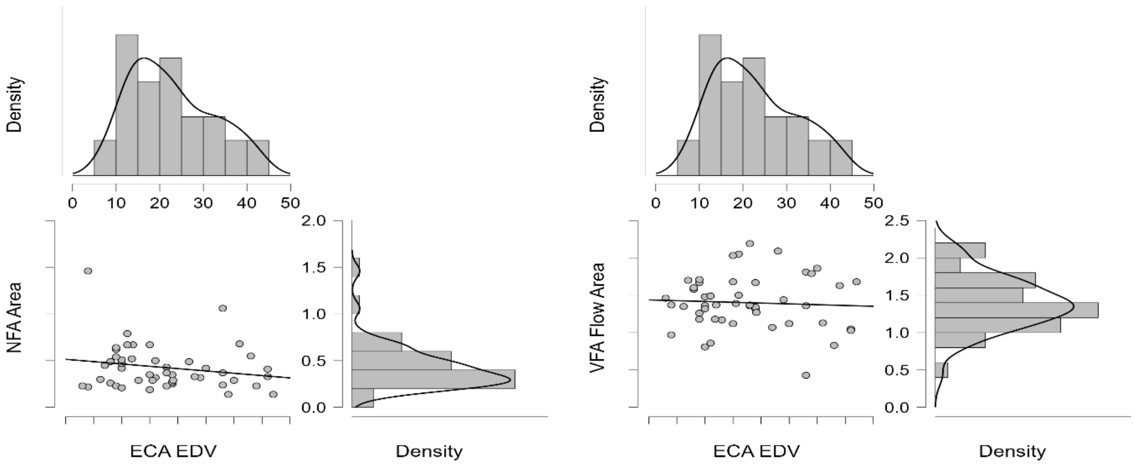

NFA: non-flow area; VFA: vascular flow area; EDV: end-diastolic velocity; ECA: external carotid artery.

Figure S5. Right eye QQ Plot for right CCA PSV and OCTA parameters (NFA Area, VFA Flow Area).

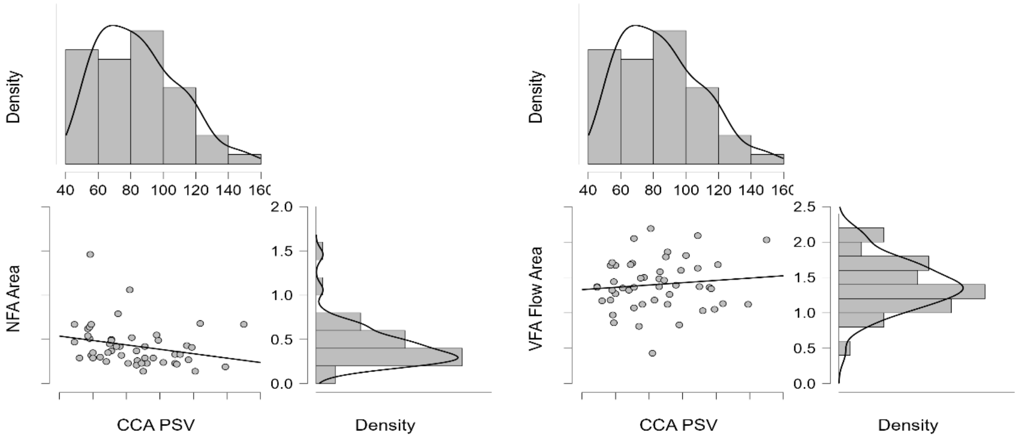

NFA: non-flow area; VFA: vascular flow area; PSV: peak-systolic velocity; CCA: common carotid artery.

Figure S6. Right eye QQ Plot for right CCA EDV and OCTA parameters (NFA Area, VFA Flow Area).

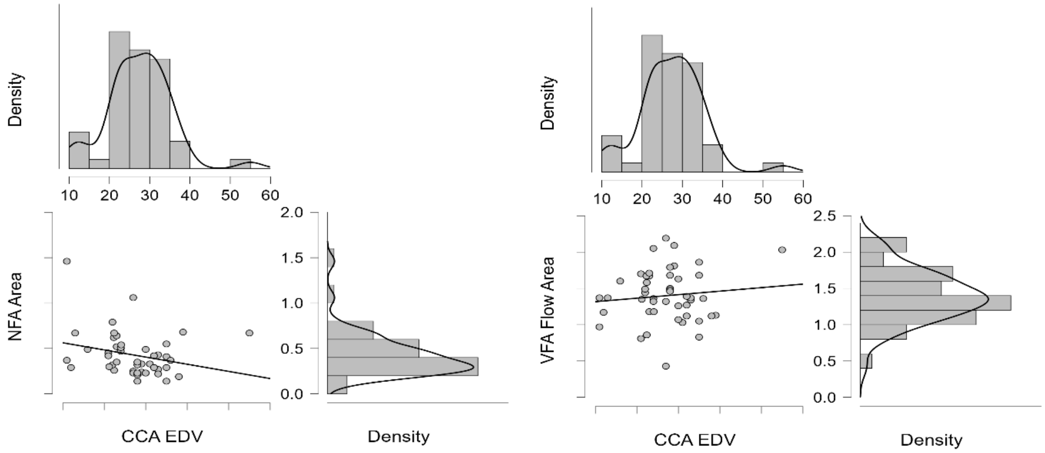

NFA: non-flow area; VFA: vascular flow area; EDV: end-diastolic velocity; CCA: common carotid artery.

Figure S7. Right eye QQ Plot for right VA PSV and OCTA parameters (NFA Area, VFA Flow Area).

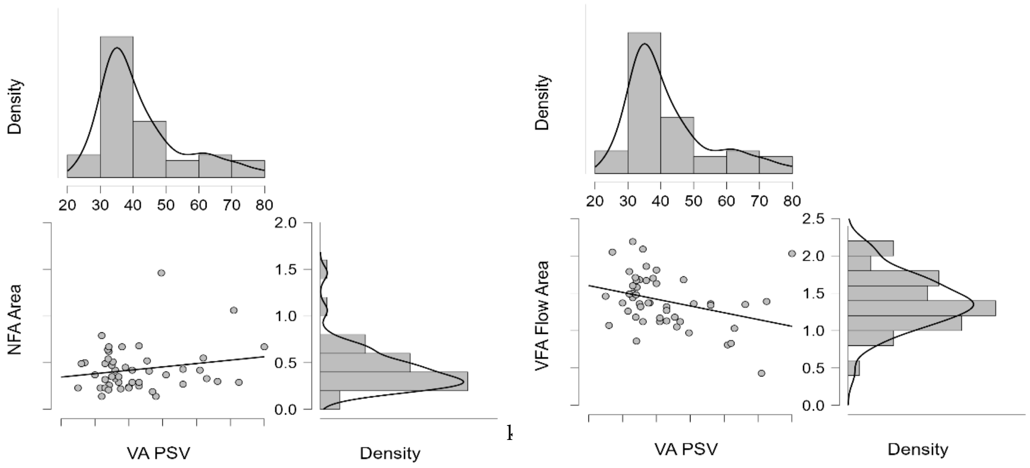

NFA: non-flow area; VFA: vascular flow area; PSV: peak-systolic velocity; VA: vertebral artery.

Figure S8. Right eye QQ Plot for right VA EDV and OCTA parameters (NFA Area, VFA Flow Area).

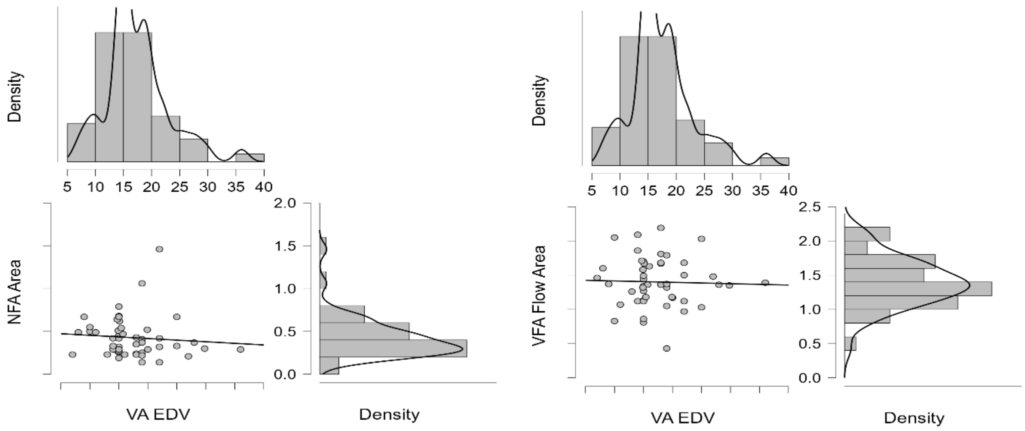

NFA: non-flow area; VFA: vascular flow area; EDV: end-diastolic velocity; VA: vertebral artery.

QQ Plots in the left eye group are displayed in Figures S9-16.

QQ Plots analyzing OCTA parameters in the left eye group and left Carotid Doppler US velocities.

Figure S9. Left eye QQ Plot for ICA PSV and OCTA parameters (NFA Area, VFA Flow Area).

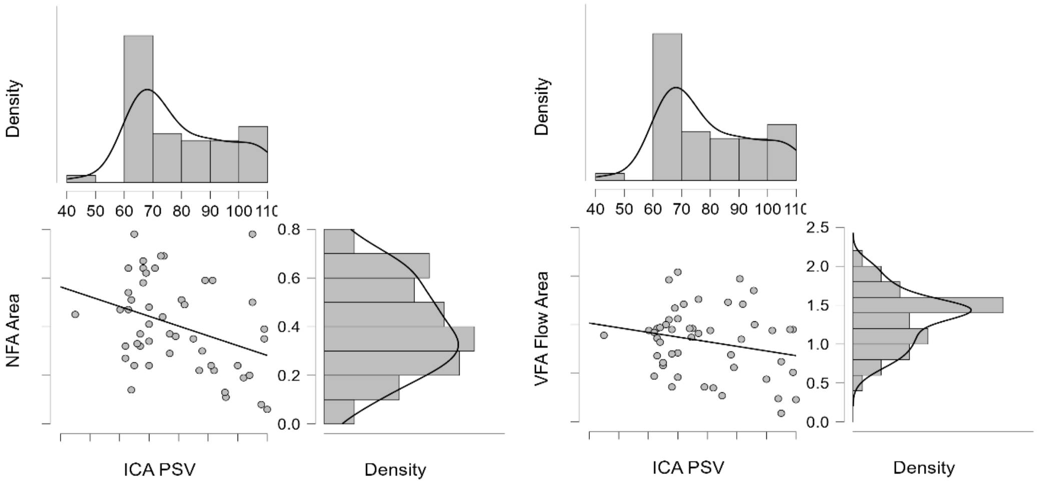

NFA: non-flow area; VFA: vascular flow area; PSV: peak-systolic velocity; ICA: internal carotid artery.

Figure S10. Left eye QQ Plot for ICA EDV and OCTA parameters (NFA Area, VFA Flow Area).

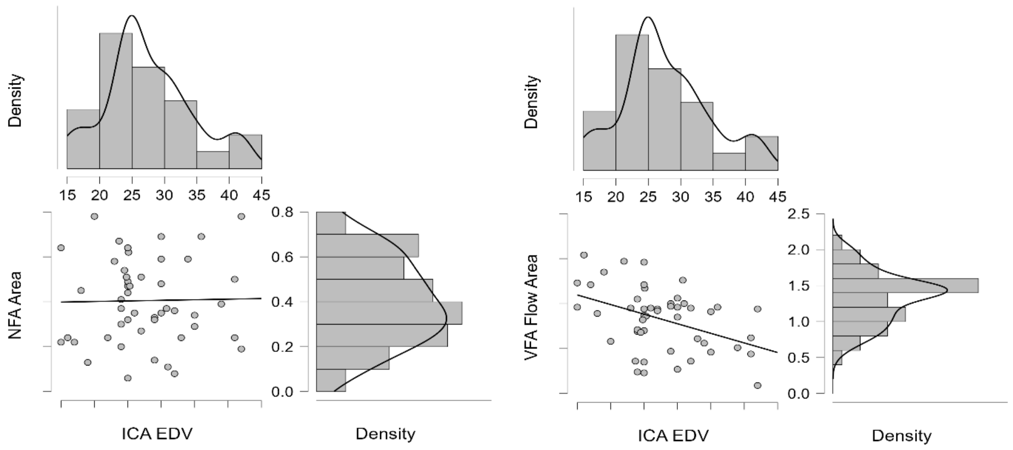

NFA: non-flow area; VFA: vascular flow area; EDV: end-diastolic velocity; ICA: internal carotid artery.

Figure S11. Left eye QQ Plot for ECA PSV and OCTA parameters (NFA Area, VFA Flow Area).

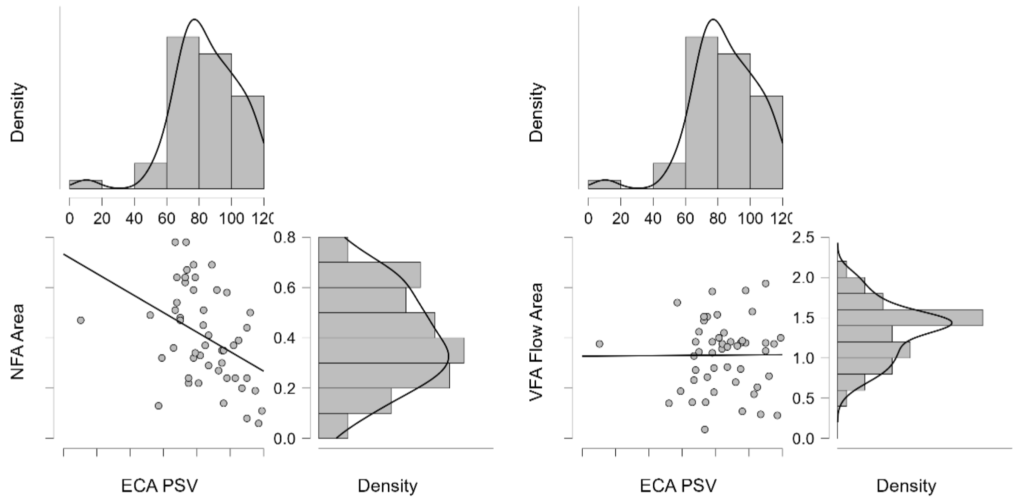

NFA: non-flow area; VFA: vascular flow area; PSV: peak-systolic velocity; ECA: external carotid artery.

Figure S12. Left eye QQ Plot for ECA EDV and OCTA parameters (NFA Area, VFA Flow Area).

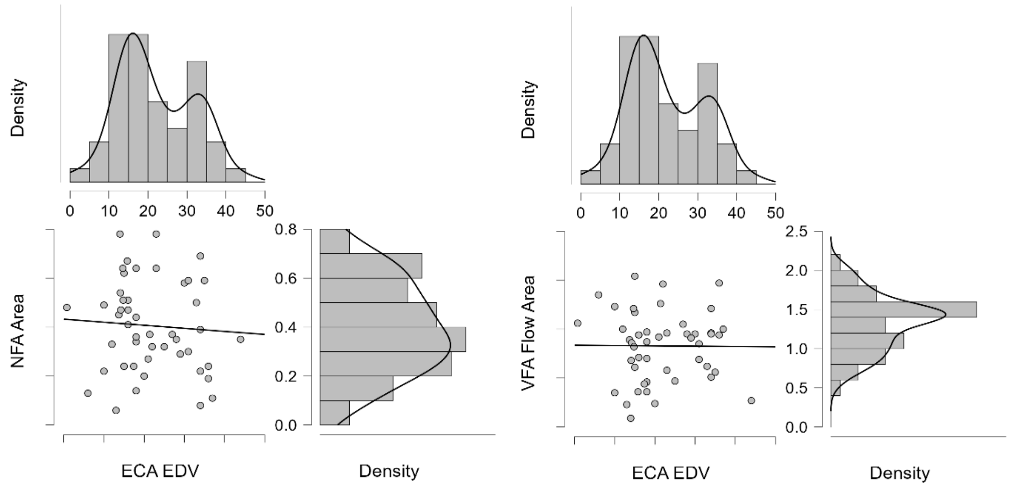

NFA: non-flow area; VFA: vascular flow area; EDV: end-diastolic velocity; ECA: external carotid artery.

Figure S13. Left eye QQ Plot for CCA PSV and OCTA parameters (NFA Area, VFA Flow Area).

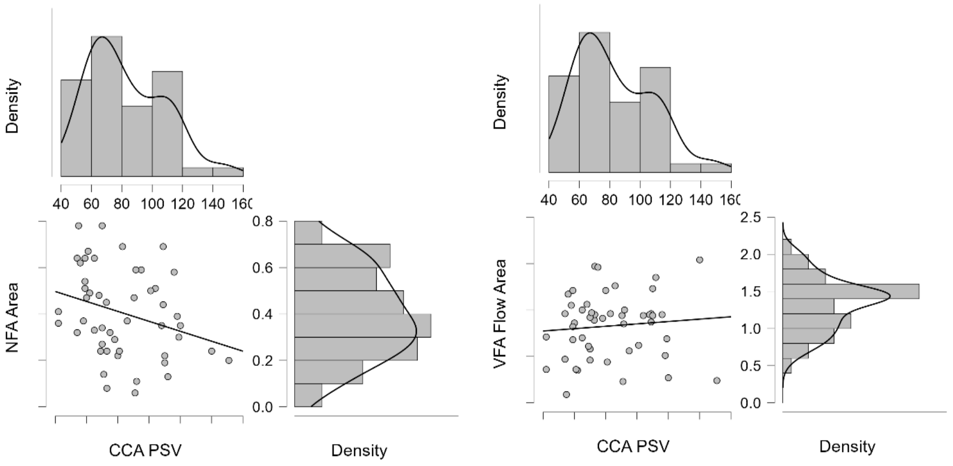

NFA: non-flow area; VFA: vascular flow area; PSV: peak-systolic velocity; CCA: common carotid artery.

Figure S14. Left eye QQ Plot for CCA EDV and OCTA parameters (NFA Area, VFA Flow Area).

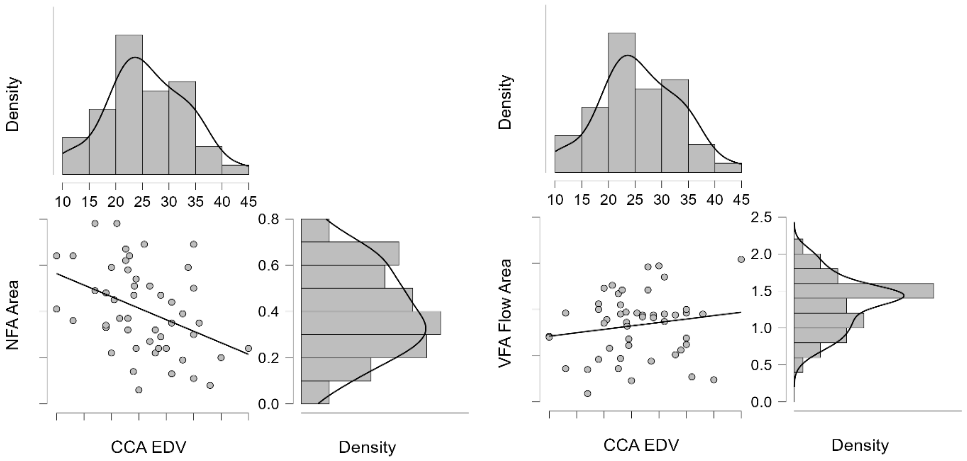

NFA: non-flow area; VFA: vascular flow area; EDV: end-diastolic velocity; CCA: common carotid artery.

Figure S15. Left eye QQ Plot for VA PSV and OCTA parameters (NFA Area, VFA Flow Area).

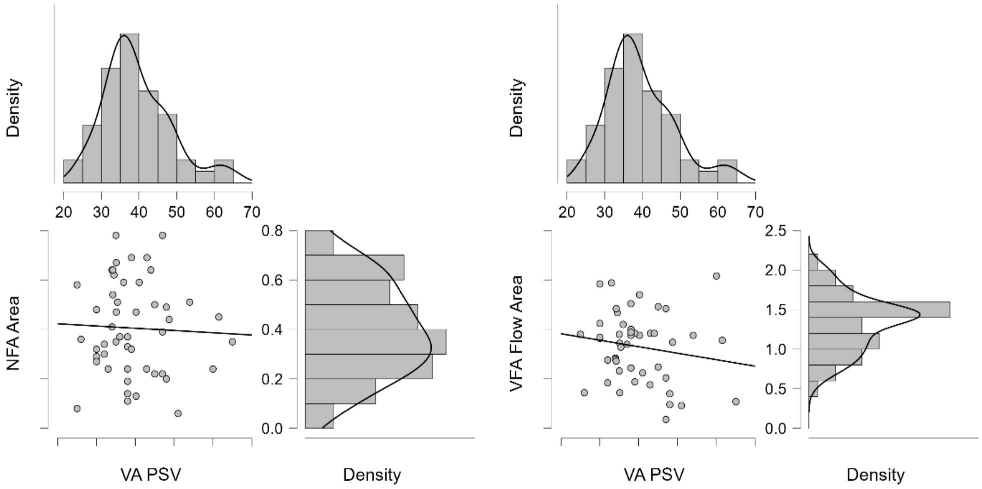

NFA: non-flow area; VFA: vascular flow area; PSV: peak-systolic velocity; VA: vertebral artery.

Figure S16. Left eye QQ Plot for VA EDV and OCTA parameters (NFA Area, VFA Flow Area).

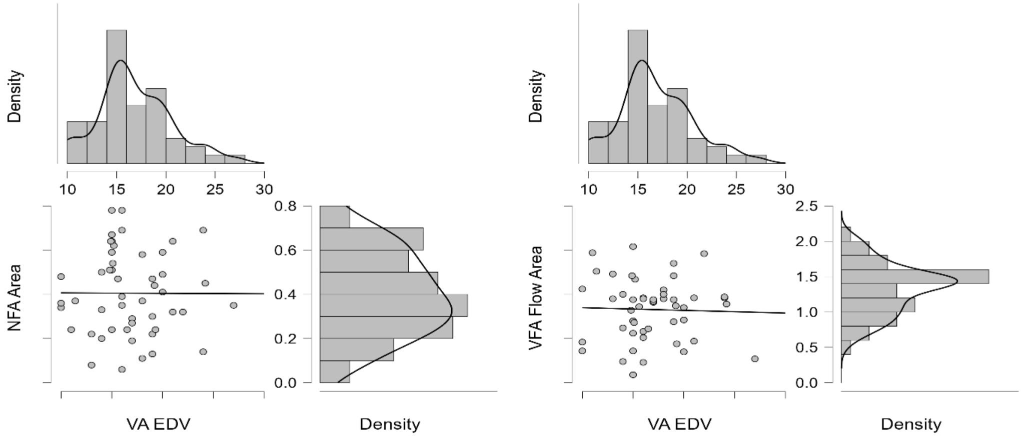

NFA: non-flow area; VFA: vascular flow area; EDV: end-diastolic velocity; VA: vertebral artery.
